# Blood/Body Fluid Exposure and Needle Stick/Sharp Injury among Nurses Working in Public Hospitals; Southwest Ethiopia

**DOI:** 10.3389/fpubh.2017.00299

**Published:** 2017-11-27

**Authors:** Yeshitila Belay Belachew, Tefera Belachew Lema, Gugssa Nemera Germossa, Yohannes Mehretie Adinew

**Affiliations:** ^1^Department of Nursing, Jimma University, Jimma, Ethiopia; ^2^Department of Population, College of Public Health and Medical Sciences, Jimma University, Jimma, Ethiopia; ^3^Department of Family Health, College of Public Health and Medical Sciences, Jimma University, Jimma, Ethiopia; ^4^College of Health Sciences and Medicine, Wolaita Sodo University, Sodo, Ethiopia

**Keywords:** occupational hazards, needle stick injury, blood/body fluids, nurses, public hospitals, Ethiopia

## Abstract

**Background:**

Every health professional around the world is at risk of blood/body fluid exposure and needle stick/sharp injury as a result of exposure to blood or body fluids and needle or sharp injuries. However, the extent of these hazards and their driving forces are not well documented in Ethiopia. Thus, the aim of this study was to assess determinants of blood/body fluid exposure and needle stick/sharp injury among nurses working in Jimma zone, southwest Ethiopia.

**Methods:**

An institution-based census was conducted among 318 nurses working in Jimma zone public hospitals from March 10 to 30, 2016. Data were collected by using pretested self-administered questionnaire. Epi info and SPSS were used for data entry and analysis, respectively. Descriptive statistics were done. Bivariate and inter multivariate logistic regression analysis was also carried out to identify predictors of occupational hazards.

**Results:**

The overall prevalence of blood/body fluid exposure and needle stick/sharp injury was found to be 249 (78.3%). Blood/body fluid exposure and needle stick/sharp injury incidents were reported by 62.6 and 58.8% of respondents, respectively. Majority of the hazards occurred during morning shift. Being male [AOR: 2.20, 95% confidence interval (CI): 1.09, 4.4], being single (AOR: 2.26, 95% CI: 1.09, 4.69), and having no training on infection prevention (AOR: 5.99, 95% CI: 3.14, 11.41) were positively associated with blood/body fluid exposure and needle stick/sharp injury; while working in chronic illness follow-up clinic (AOR: 0.19, 95% CI: 0.05, 0.71) showed negative association at *p* value of 0.05.

**Conclusion:**

Prevalence of blood/body fluid exposure and needle stick/sharp injury was high among the nurses. The safety of nurses depends directly on the degree to which nurses can identify and control the varied occupational hazards specific to jobs. Thus, working unit specific safety precautions and basic infection prevention in-service training might improve nurses’ safety practice and thereby decrease the on job hazard.

## Introduction

Occupational hazard is any condition of a job that can produce a negative effect on peoples’ health, either immediately or over time (1). Blood and body fluid exposures and needle stick injuries have been recognized as one of the occupational hazards among health-care workers (HCWs) (2, 3). Needle stick injury (NSI) is occupational exposure to patients’ body fluids when a needle or other sharp object penetrates the skin and it is interchangeable with sharps injury (4). Occupational hazard places HCWs at risk for numerous blood-borne infections, most importantly human immunodeficiency virus, hepatitis B and C viruses (5, 6).

Despite advances in understanding and control of infections, occupational blood and body fluids exposure and NSIs continue to be the major worldwide public health problems (7) and serious concern for around 35 million HCWs globally (8). Biological, chemical, and mechanical hazards are affecting over 20 million HCW annually. Three million health professionals are estimated to be exposed to blood and body fluids due to needle stick or sharps injuries daily (9) and over 90% of the cases occur in resource constrained countries according to World Health Organization (8). Utilization of safe needle devices can avoid three-fourth of these hazards (9).

Nurses are the major health-care providers in the hospital with more exposure to blood and body fluids (7), as most of their NSIs, the most prevalent occupational hazard, incidents involve devices like hollow-bore needles that are very efficient at transmitting pathogens (10, 11). Around two-thirds of disease sero-conversions following NSIs occur among nursing staff (12). The problem is expected to be more devastating in developing countries like Ethiopia where health setup is poor (13).

Information about the prevalence of occupational hazards and its determinants is crucial for occupational health planning. But, little is known about the prevalence of occupational hazards in the study area. Therefore, this study was aimed to assess the prevalence of blood/body fluid exposure and needle stick/sharp injury and associated factors among nurses working in Jimma zone public hospitals, southwest Ethiopia.

## Materials and Methods

### Study Area and Period

An institution-based census was conducted among 318 nurses working in three public hospitals in Jimma zone. The hospitals were Shenen Gibe, Limu Genet, and Jimma University Specialized Hospital and all participants have experience of ≥6 months. Jimma is located at a distance of 352 km from the capital Addis Ababa in the southwest direction. The zone has a total population of 2,486,155; with an area of 15,568.58 square kilometer (14).

### Data Collection Instrument and Procedure

Data were collected using a pretested and structured self-administered questionnaire adopted from previous studies (15–17). Questionnaires were prepared and administered in English as the respondents were professional nurses. The questionnaire consisted of two parts. The first part contains eight questions and was used to assess the sociodemographic characteristics of respondents; the second part includes 15 major items and 47 sub-items for measuring blood/body fluid exposure and needle stick/sharp injury and work-related factors. Nine data collectors guided by one supervisor collected the data from March 10 to 30, 2016.

### Data Quality Assurance

Data quality was controlled by giving training and appropriate supervision for data collectors. The overall supervision was carried out by the principal investigator. The questionnaire was pretested on 18 (5%) nurses working in Woliso hospital. Based on the pretest analysis, appropriate modifications were made to the questionnaire before the actual data collection.

### Data Processing and Analysis

The filled questionnaires were entered into Epi Info version 3.5.4 after checking for completeness and then exported to SPSS version 20 for further analysis. Descriptive statistics were done. Bivariate and inter multivariate logistic regression models were also carried out. Sex, marital status, age category, training on infection prevention, position in the hospital, and working unit were the variables entered to the multivariate regression. Odds ratios and their 95% confidence intervals (CI) were computed and variables with *p*-value less than 0.05 were considered significant.

### Operational Definitions

#### Body Fluid

Body fluid includes vomits, urine, sputum, saliva, amniotic fluid, exudative fluids from burns/lesions, and cerebrospinal fluid.

## Results

### Sociodemographic Characteristics of Study Participants

**Figure 1:**
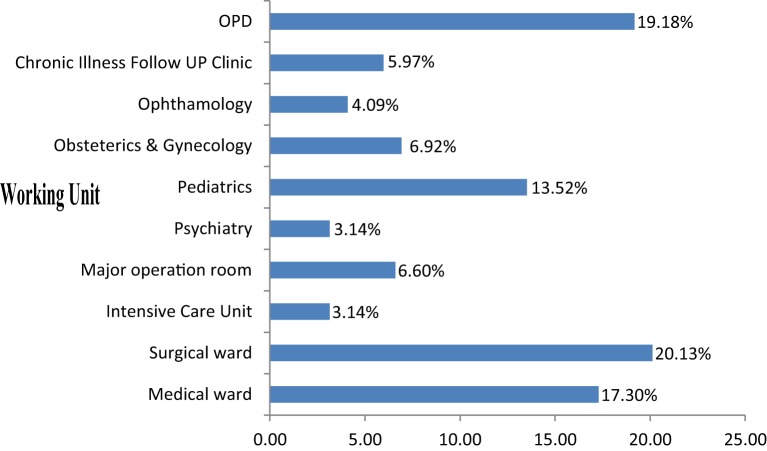
Distribution of nurses working in each unit in Jimma zone public hospitals, southwest Ethiopia, 2016.

Out of the expected 341 nurses in selected hospitals, 318 agreed to participate in the study, yielding a response rate of 93.3%. The mean age of the participants was 27.9 years (SD ± 6.84). Male and single respondents accounted for 50.6 and 54.7%, respectively. More than half 173 (60.7%) of participants were diploma holders. Majority 229 (75.2%) of them had less than 5 years of work experiences (Table 1). One-fifth (20.1%) of respondents identified surgical ward as their working unit; followed by outpatient department (19.2%) and medical ward (17.3%) (Figure 1).

**Table 1:** Sociodemographic characteristics of nurses working in Jimma zone public hospitals, southwest Ethiopia, 2016.

Characteristics	*N* = 318	*n*	%
Working hospital	Jimma University Specialized Hospital	273	85.8
	Shenen Gibe Hospital	20	6.3
	Limu Genet Hospital	25	7.9
Sex	Male	161	50.6
	Female	157	49.4
Age group	≤24 years	106	33.3
	25–29 years	150	47.2
	≥30 years	62	19.5
Marital status	Married	136	42.8
	Single	174	54.7
	Divorced	7	2.2
	Widowed	1	0.3
Educational qualification	Diploma	193	60.7
	BSc	125	39.3
Work experiences in years	<5 years	239	75.2
	≥5 years	79	24.8
Position/title in the hospital	Staff nurse	283	89
	Head nurse	28	8.8
	Supervisor nurse	4	1.3
	Matron nurse	3	0.9

### Prevalence of Blood/Body Fluid Exposure and Needle Stick/Sharp Injury

The overall prevalence of the hazard was found to be 249 (78.3%). Around two-third 187 (58.8%) of the participants reported sharp injuries; syringe needle (58.8%) and broken ampoule (43.3%) were the dominants. From the reported sharp injuries, 86 (46.0%) occurred when the needle was used for an injection and 78 (41.7%) occurred during ampoule breaking. Out of the 187 nurses exposed to sharp injuries, 72 (38.5%) had experienced it twice (Table 2).

**Table 2:** Prevalence of needle/sharp injuries in Jimma zone public hospitals, southwest Ethiopia, 2016.

		Yes (%)	No (%)
Needle stick/sharp injury		187 (58.8%)	131 (41.2%)
Types of needle and sharp materials	Syringe needle	110 (58.8%)	77 (41.2%)
	Suturing needle	41 (21.9%)	146 (78.1%)
	Butterfly needle	22 (11.8%)	165 (88.2%)
	IV needle	47 (25.1%)	140 (74.9%)
	Insulin syringe	7 (3.7%)	180 (96.3%)
	Lancet	18 (9.6%)	169 (90.4%)
	Surgical blade	34 (18.2%)	153 (81.8%)
	Brocken ampoule	81 (43.3%)	106 (56.7%)
Types of procedures related to needle stick and sharp injuries	Injection	86 (46.0%)	101 (54.0%)
	Breaking ampoule	78 (41.7%)	109 (58.3%)
	Suturing/sewing	45 (24.1%)	142 (75.9%)
	Blood drawing	33 (17.6%)	154 (82.4%)
	Needle recapping after use	14 (7.5%)	173 (92.5%)
	Vein puncture	56 (29.9%)	131 (70.1%)
	Misplaced needle	19 (10.2%)	168 (89.8%)
	Disposing	33 (17.6%)	154 (82.4%)

Almost two-third 199 (62.6%) of the participants were exposed to blood/body fluids. Of the reported exposures to blood/body fluids, 177 (88.9%) involved splashes of blood; whereas 81 (40.7%) were urine. Most of the exposures occurred during blood drawing 102 (51.3%), vein puncture 94 (47.2%), and injection 60 (30.2%). About one-third 59 (29.6%) of the nurses were exposed twice (Table 3).

**Table 3:** Exposure to blood/body fluids among nurses in Jimma zone public hospitals, southwest Ethiopia, 2016.

		Yes (%)	No (%)
Blood and body fluid exposures		199 (62.6%)	119 (37.4%)
Types of blood and body fluids^a^	Blood	177 (88.9%)	22 (11.1%)
	Vomit	66 (33.2%)	133 (66.8%)
	Saliva	54 (27.1%)	145 (72.9%)
	Urine	81 (40.7%)	118 (59.3%)
	Amniotic fluid	7 (3.5%)	192 (96.5%)
	Exudative fluids from burns/lesions	30 (15.1%)	169 (84.9%)
	Cerebrospinal fluid	4 (2.0%)	195 (98.0%)
Types of procedure related to blood and body fluid exposures^a^	Injection	60 (30.2%)	139 (69.8%)
	Blood drawing	102 (51.3%)	97 (48.7%)
	Vein puncture	94 (47.2%)	105 (52.8%)
	Recapping of needle after use	12 (6.0%)	187 (94.0%)
	Misplaced needle	12 (6.0%)	187 (94.0%)
	Disposing	51 (25.6%)	148 (74.4%)

*^a^Multiple answers possible (percent do not add 100)*.

Though 232 (73%) of the respondents have no formal training on infection prevention, almost all (99.1%) of them reported utilization of personal protective devices on duty. Regarding work load, around one-fourth (23.6%) of nurses were attending 10 patients per day, while 44 (13.8%) were attending 15 on average.

Increased exposures to occupational hazards were reported during the morning shift; 111 (59.4%) sharp/needle stick injuries and 130 (65.3%) exposure to blood/body fluids (Table 4).

**Table 4:** Distribution of occupational hazards against working shift among nurses working in Jimma zone public hospitals, southwest Ethiopia, 2016.

Variables	Types of occupational hazards			
Working shift^a^	Needle stick/sharp injuries		Blood/body fluid exposures	
	Frequency (*n*)	%	Frequency (*n*)	%
Morning	111	59.4	130	65.3
Evening	64	34.2	79	39.7
Night	101	54.0	103	51.8

*^a^Multiple answers possible (percent do not add 100)*.

### Factors Associated With Occupational Hazards

Males and single participants conceded increased occupational hazards; while having training on infection prevention and working in chronic illnesses follow-up ward were protective. Age and administrative position in the hospital did not show significant association with occupational hazards. Single respondents were about two times more likely to have occupational hazards than ever married ones (AOR: 2.26, 95% CI: 1.09, 4.69). The odds of exposure was two times higher for male nurses than their female counterparts (AOR: 2.20, 95% CI: 1.09, 4.40). Respondents who did not take any training on infection prevention were six times more likely to have occupational hazards than those who did (AOR: 5.99, 95% CI: 3.14, 11.41). Occupational hazard was found to have significant association with working department where nurses working in chronic illnesses follow-up clinic had 81% less exposure as compared to nurses working in surgical ward (AOR: 0.190, 95% CI: 0.05, 0.71) (Table 5).

**Table 5:** Bivariate and multivariate logistic regression model among nurses working in Jimma zone public hospitals, south west Ethiopia, 2016.

Variables		Occupational hazards		COR [95.0% confidence interval (CI)]	AOR (95.0% CI)
		Yes (*N* = 249)	No (*N* = 69)		
Sex	Female	112	45	1	
	Male	137	24	2.29 (1.32, 3.99)	2.20 (1.09, 4.40)*
Marital status	Ever married	99	45	1	
	Single	150	24	2.84 (1.63, 4.96)	2.26 (1.09, 4.69)*
Age category	≥30 years	43	19	1	
	≤24 years	86	20	1.90 (0.92, 3.93)	
	25–29 years	120	30	1.77 (0.90, 3.46)	
Training on infection prevention	Yes	46	40	1	
	No	203	29	6.09 (3.42, 10.82)	5.99 (3.14, 11.41)*
Position in the hospital	Clinical staff manager	20	15	1	2.20 (0.89, 5.42)
	Clinical staff nurse	229	54	3.18 (1.53, 6.61)	
Working unit	Surgical ward	52	12	1	
	Medical ward	47	8	1.36 (0.51, 3.60)	
	Intensive care unit	7	3	0.54 (0.12, 2.39)	
	Major operation room	17	4	0.99 (0.28, 3.45)	
	Psychiatry	7	3	0.54 (0.12, 2.39)	
	Pediatrics ward	39	4	2.25 (0.67, 7.51)	
	Obstetrics and gynecology ward	18	4	1.04 (0.29, 3.63)	
	Ophthalmology unit	9	4	0.52 (0.14, 1.97)	
	Chronic illness follow-up clinic	9	10	0.208 (0.07, 0.62)	0.19 (0.051, 0.710)*
	OPD	44	17	0.60 (0.26, 1.39)	

*Statistically significant.

*NSIs, needle stick injuries; COR, crude odds ratio; AOR, adjusted odds ratio; OPD, outpatient department*.

## Discussion

Despite advances in understanding and control of infections, occupational hazards continued to be the major worldwide public health problem (7). It is the most important problem for HCWs as it increases the risk of infection by exposing them to more than 20 different blood-borne pathogens (18). The problem is more devastating in developing countries like Ethiopia, where health setup is poor (13). Nurses emerge as the staff group reporting the highest proportion of such hazards. Thus, this study was aimed to assess the prevalence of occupational hazards and associated factors among nurses working in Jimma zone public hospitals.

The overall prevalence of occupational hazards in the study area was 78.3%. Blood/body fluid exposure during the last 6 months was 62.6% while needle stick/sharp injuries during the same period was found to be 58.8%; implying that, blood/body fluid exposures and NSIs are common occupational hazards to the participants. This finding is in line with a study from Turkey where the prevalence was 57% (19). Majority of NSIs among nurses occurred by syringe needle as most procedures in a clinical setting involve administering intravenous/intramuscular injections or the drawing of blood which is almost comparable with study done in Saudi Arabia (63%) (20).

Risks of sharp injuries varied between different working units. In this study, 21% of exposures occurred in the surgical ward. This result is consistent with the study finding from tropical Australian hospital (23.9%) (12). Prevalence of sharp injuries in intensive care unit (3.1%) was also comparable with the finding of study conducted in British Columbia (3.0%) (21).

The proportion of nurses experienced blood exposure in this study is similar to that of southern Iran in which blood was the most frequent contaminant (87%). The exposure most commonly occurs during inserting or removing intravenous lines and the high prevalence might be due to insufficient number of nurses, lack of adherence to standard precautions, and improper disposal of medical wastes (22).

Sex of respondents was also significantly associated with occupational hazards; the odds of exposure were two times higher among male nurses than females. This is consistent with the report from the duke health and safety surveillance system (5). The possible explanations might be men are less likely to use universal precautions but further studies are warranted to identify exposure differences, especially in nursing staff who perform similar tasks.

Marital status was found to have significant association with occupational hazards. Single participants had increased risk of encountering occupational hazards compared to ever married ones. The possible explanation might be married nurses feel more responsible than the single ones which increase their chance of adhering to universal precautions but further studies are required to produce solid evidences.

Training on infection prevention was the other predictor of occupational hazards. Nurses who had no training on infection prevention were six times more exposed to risk of occupational hazards than those who had. This result is in line with the study finding from sub-Saharan Africa (23). In this study 73% of the respondents had never been trained on infection prevention and safety precautions; which shows poor culture of the health facilities in practicing safety first principle. This might be the reason behind the high prevalence of blood/body fluid exposure and needle stick/sharp injury; as training activities in nursing increases the chance of the trainees to get up-to-date information about infection prevention mechanisms (24), promote job satisfaction, increase retention of nurses, and even enable continued provision of quality care (25).

Nurses working in chronic illnesses follow-up clinic were less likely to have occupational hazards than those nurses working in surgical ward. similar finding was obtained in tropical Australian hospital (12) and China (26) where nurses working in maternity/neonatal units were less likely to have occupational hazards compared to nurses working in medical or surgical wards.

### Limitations of the Study

This study has shared the limitations of cross-sectional studies, the difficulty of determining causal relationships between variables. The instrument was prepared in English assuming respondents can understand the language and this may possibly cause some miss understanding in some words or terminology.

## Conclusion

The prevalence of occupational hazard among nurses was found to be high. Blood/body fluid exposure accounts greater number than needle stick/sharp injuries. Being male and single was positively associated with occupational hazards; while having training on infection prevention and working in chronic illness follow-up clinic showed negative association. Working unit specific safety precautions and basic infection prevention in-service training may help the nurses to practice safety first principle and there by decrease blood/body fluid exposure and needle stick/sharp injuries as the safety of nurses themselves and subsequently, that of their patients, depend directly on the degree to which nurses can identify and control the varied occupational hazards specific to jobs.

## Consent to Publish

Written consent was obtained from the respondents to publish the interview.

## Availability of Data and Materials Section

Data supporting this finding are available.

## Ethics Statement

Ethical clearance was obtained from Jimma University, College of public health and Medical sciences, institutional review board. A formal letter of cooperation was written to the hospitals. Written consent was obtained from each study participant.

## Author Contributions

YBB wrote the proposal, participated in data collection, analyzed the data, and drafted the paper. BTL and GNG approved the proposal with some revisions, participated in data analysis. YMA participated in proposal development, data analysis, and wrote the manuscript. All authors read and approved the final manuscript.

## Conflict of Interest Statement

The authors declare that the research was conducted in the absence of any commercial or financial relationships that could be construed as a potential conflict of interest.

## Abbreviations

AOR, adjusted odds ratio; COR, crude odds ratio; HCWs, health-care workers; HIV, human immunodeficiency virus; NSI, needle stick injury; OPD, outpatient department; WHO, World Health Organization.
